# Kin selection of time travel: the social evolutionary causes and consequences of dormancy

**DOI:** 10.1098/rspb.2023.1247

**Published:** 2023-09-13

**Authors:** Kalyani Z. Twyman, Andy Gardner

**Affiliations:** School of Biology, University of St Andrews, Greenside Place, St Andrews KY16 9TH, UK

**Keywords:** kin selection, dormancy, dispersal, diapause, altruism, population viscosity

## Abstract

A basic mechanism of kin selection is limited dispersal, whereby individuals remain close to their place of origin such that even indiscriminate social interaction tends to modify the fitness of genealogical kin. Accordingly, the causes and consequences of dispersal have received an enormous amount of attention in the social evolution literature. This work has focused on dispersal of individuals in space, yet similar logic should apply to dispersal of individuals in time (e.g. dormancy). We investigate how kin selection drives the evolution of dormancy and how dormancy modulates the evolution of altruism. We recover dormancy analogues of key results that have previously been given for dispersal, showing that: (1) kin selection favours dormancy as a means of relaxing competition between relatives; (2) when individuals may adjust their dormancy behaviour to local density, they are favoured to do so, resulting in greater dormancy in high-density neighbourhoods and a concomitant ‘constant non-dormant principle’; (3) when dormancy is constrained to be independent of density, there is no relationship between the rate of dormancy and the evolutionary potential for altruism; and (4) when dormancy is able to evolve in a density-dependent manner, a greater potential for altruism is expected in populations with lower dormancy.

## 1. Introduction

Kin selection is a fundamental force shaping social evolution [1]. One of the three basic mechanisms that give rise to kin selection is limited dispersal—or ‘population viscosity’—whereby individuals remain close to their place of origin such that even indiscriminate social interactions tend to lead to fitness consequences for their genealogical kin [1,2]. Accordingly, the causes and consequences of dispersal have received an enormous amount of attention in the social evolution literature [3,4]. Concerning the social evolutionary causes of dispersal, Hamilton & May [5] showed that kin selection favours dispersal as a means of relaxing competition between kin for reproductive resources. Later, Crespi & Taylor [6] showed that, on account of kin competition being greatest in high-density neighbourhoods, such neighbourhoods are predicted to exhibit a higher rate of dispersal and, in fact, the absolute number of non-dispersing individuals in a neighbourhood is expected to be completely independent of local density—the ‘constant non-disperser principle’.

Concerning the social evolutionary consequences of dispersal, Taylor [7] showed that, in the simplest model scenario, the two opposing effects of dispersal in reducing relatedness and relaxing kin competition exactly cancel each other such that there is no net impact of the rate of dispersal on the evolutionarily favoured level of altruistic behaviour. This invariance result has stimulated a huge amount of theoretical—and, increasingly, empirical—investigation into the interplay of relatedness and kin competition in a variety of ecological and demographic settings [4]. Recently, Kanwal & Gardner [8] showed that if individuals can condition their probability of dispersal on local density—following Crespi & Taylor's ‘constant non-disperser principle’—the link between dispersal and kin competition is broken such that a higher level of altruism is, in fact, favoured in viscous populations.

The above account of the social evolutionary causes and consequences of dispersal has concerned the dispersal of individuals through space. Yet, similar logic should apply to dispersal of individuals through time (e.g. dormancy). Dormancy has long been conceptualized as analogous to dispersal, with these two life-history traits often sharing similar physiology as well as being driven by the same or similar selection pressures, including the adaptive rationale of serving to ease kin competition [8–13]. Several theoretical studies of delayed germination in seeds have suggested that, like dispersal, dormancy may serve to reduce the impact of local competition among relatives in stable habitats [11,14,15]. Additionally, theoretical analysis of the joint evolution of dormancy and dispersal in seeds reveals that a higher level of each of these traits reduces selection for the other, implying that dormancy and dispersal are alternative solutions to the same problem [11,12]. However, the analogy between the evolution of dormancy and the evolution of dispersal has been somewhat obscured because these analytical treatments have assumed that, while a dispersing individual travels an effectively infinite distance in space, a dormant individual travels only one generation into the future. Moreover, the analogy between dormancy and dispersal in terms of the impact upon the evolution of altruistic behaviour remains completely unexplored.

Here, we explore the analogy between dispersal in space and dispersal in time by investigating the social evolutionary causes and consequences of dormancy of arbitrary duration. We perform a series of kin-selection analyses in the context of the infinite island model of population structure to determine how kin selection drives the evolution of density-independent and density-dependent dormancy and how density-independent and density-dependent dormancy then drive the evolution of altruistic behaviour. This yields direct analogues of Hamilton & May's [5] evolution of dispersal result, Crespi & Taylor's [6] ‘constant non-disperser principle’, Taylor's [7] dispersal invariance result, and Kanwal & Gardner's [8] recovery of the altruism-promoting effect of population viscosity under density-dependent dispersal—but for the dispersal of individuals through time.

## 2. Results

### (a) Kin selection promotes dormancy

Hamilton & May [5] found that competition for reproductive resources among relatives strongly promotes the evolution of dispersal as a means of separating kin. In their simple model scenario, in which a single breeder produces a clutch of clonal offspring in each patch, the resulting kin competition is so strong that more than half of these offspring are favoured to disperse even as the cost of dispersal approaches lethality. Allowing multiple breeders in each patch reduces relatedness and leads to a lower rate of dispersal being evolutionarily favoured, yet kin competition nevertheless remains a substantial selective force [16,17]. Here, we perform an analogous treatment of the evolution of dormancy.

We assume an infinite patch-structured population in which each patch contains *n* asexual, haploid individuals. Each of these individuals produces a large number of offspring and then dies. Each of the offspring then disperses to a randomly chosen patch elsewhere in the population with probability *d* or else remains in their natal patch with probability 1 − *d*. Non-dispersing individuals may go dormant, in which case they die with probability *c* or else exit dormancy *t* generations later, with *t* being a random variable with probability distribution *ϕ*(*t*) defined over a set of positive integers. The core results of our analysis obtain without making any further assumptions as to the shape of *ϕ*(*t*), although we do make use of the special case of infinitely long dormancy for the purpose of illustration and to facilitate synthesis with the existing literature—these instances are explicitly highlighted below. Following both dispersal and entry to and exit from dormancy in each generation, *n* non-dormant individuals are chosen at random in each patch to become breeders, with all the other non-dormant individuals perishing, such that the overall population size remains constant across generations.

Applying the Taylor–Frank [18] method of kin selection analysis, we find that the condition for natural selection to favour an increase in the probability of entering dormancy is

2.1 $$\begin{array}{rl} & -c+\frac{\left(1-d)(1-\delta \right)}{1-c(1-d)\delta }r+\frac{(1-d)(1-c)\delta }{1-c(1-d)\delta }R-(1-c)\frac{\left(1-d)(1-\delta \right)}{1-c(1-d)\delta }R\\ & \;-(1-c)\frac{(1-d)(1-c)\delta }{1-c(1-d)\delta }\rho >0, \end{array}$$

where δ is the population-average probability with which an individual enters dormancy, *r* is the average relatedness of patchmates born in the same generation, *R* is the average relatedness of an individual exiting dormancy to patchmates born at that time, and ρ is the average relatedness of two individuals exiting dormancy in the same patch at the same time (see electronic supplementary material for details).

Expression (2.1) is a form of Hamilton's rule [1,19,20], and the terms on the left-hand side admit an inclusive fitness interpretation. Specifically, an increase in dormancy: (i) incurs a survival cost -*c* for the focal individual; (ii) reduces resource competition for the proportion (1 − *d*)(1 − *δ*)/(1 − *c*(1 − *d*)*δ*) of patch residents who have neither dispersed nor entered dormancy, and who are related to the focal individual by *r*; (iii) reduces resource competition for the proportion (1 − *d*)(1 − *c*)*δ*/(1 − *c*(1 − *d*)*δ*) of patch residents who have just exited dormancy, and who are related to the focal individual by *R*; (iv) increases resource competition for the proportion (1 − *d*)(1 − *δ*)/(1 − *c*(1 − *d*)*δ*) of patch residents in the future generation who have neither dispersed nor entered dormancy, and who are related to the focal individual by *R*, to the extent 1-*c* that the focal individual survives dormancy; and (v), increases resource competition for the proportion (1 − *d*)(1 − *c*)*δ*/(1 − *c*(1 − *d*)*δ*) of patch residents who have exited dormancy in the same generation as the focal individual, who are related to the focal individual by *ρ*, to the extent 1 − *c* that the focal individual survives dormancy. Note that, unlike in infinite island-model analyses of the evolution of dispersal, in which a dispersing individual never encounters relatives in her new patch, individuals undergoing a finite period of dormancy may encounter relatives when they exit dormancy, yielding kin-selected costs (4th and 5th terms of expression (2.1)) in addition to the kin-selected benefits (2nd and 3rd terms) of dormancy.

If the convergence stable [21] probability of dormancy takes an intermediate value, then this may be found by setting the left-hand side of expression (2.1) equal to zero and solving for *δ* = δ*, which yields

2.2 $$\delta^{*}\;=\;\frac{r-(c/1-d)-(1-c)R}{r-c^{2}-2(1-c)R+{\left(1-c\right)}^{2}\rho }.$$

The three coefficients of relatedness appearing in expression (2.2) are not independent model parameters, but are instead jointly determined by the demography of the population. For example, the relatedness of two individuals born on the same patch at the same time is given by

2.3 $$\begin{array}{rl} r & =\frac{1}{n}\\ & \;+\frac{n-1}{n}{\left(\frac{\left(1-d)(1-c\;\delta \right)}{1-c\;(1-d)\;\delta }\right)}^{2}\left(\begin{array}{c} \;{\left(\frac{\left(1-\delta \right)}{1-c\;\delta }\right)}^{2}r\\ +2\left(\frac{\left(1-\delta \right)}{1-c\;\delta }\right)\left(\frac{(1-c)\;\delta \;}{1-c\;\delta }\right)R\\ +{\left(\frac{(1-c)\;\delta }{1-c\;\delta }\right)}^{2}\rho \end{array}\right). \end{array}$$

That is: with probability 1/*n*, two individuals born in the same patch at the same time share the same mother, and are thus related to each other by 1; and with probability (*n* − 1)/*n*, they have different mothers and their relatedness to each other is equal to that of their mothers. With probability ((1 − *d*)(1 − *cδ*)/(1 − *c*(1 − *d*)*δ*))^2^, both mothers were locals, in which case: with probability ((1 − *δ*)/(1 − *cδ*))^2^ neither mother had undergone dormancy, and hence they were related by *r*; with probability 2((1 − *δ*)/(1 − *cδ*))((1 − *c*)*δ*/(1 − *cδ*)) one mother had undergone dormancy and the other had not, and hence they were related by *R*; and with probability ((1 − c) *δ*/(1 − *cδ*))^2^ both mothers had undergone dormancy, and hence they were related by *ρ*.

It is not possible, in general terms, to give the relatedness coefficients as explicit functions of the model's demographic parameters (i.e. *n*, *c*, *d*, *δ* and *ϕ*(*t*)). However, explicit solutions for the convergence stable probability of dormancy may be obtained for special cases. For example, if dormancy is of fixed duration—whereby everyone exiting dormancy at a given time would also have entered it in the same generation—then the convergence stable probability of dormancy is maximally one half (i.e. δ* = ½ if *ρ* = *r* and *c* = 0). This reflects how same-generation patchmates are, in this scenario, most efficiently separated by having half of them competing in the present and the other half competing in the future, with any deviation from one half leading to an exacerbation of kin competition. This also explains why the probability of dormancy does not exceed one-half in scenarios in which the duration of dormancy is just one generation [11,12,14], and extends the principle to dormancy of any fixed duration.

Conversely, if dormancy is of variable duration, then, in the limit of infinitely long dormancy—whereby individuals exiting from dormancy never encounter their genetic relatives (i.e. such that *R* = *ρ* = 0)—expression (2.3) reduces to

2.4 $$r=\;\frac{{\left(1-c\;(1-d)\;\delta \right)}^{2}}{n\;{\left(1-c\;(1-d)\;\delta \right)}^{2}-(n-1)\;{\left(1-d\right)}^{2}\;{\left(1-\delta \right)}^{2}}.$$

and expression (2.2) reduces to

2.5 $$\begin{array}{rl} \delta^{*} & =\frac{(1-d)r-c}{\left(1-d)(r-c^{2}\right)}\\ & =\frac{2cn((1-d)-c)+(1-d-c)\left(1-\sqrt{1+4c^{2}(n-1)n}\right)}{2cn(1-c^{2})(1-d)}. \end{array}$$

**Figure 1.**
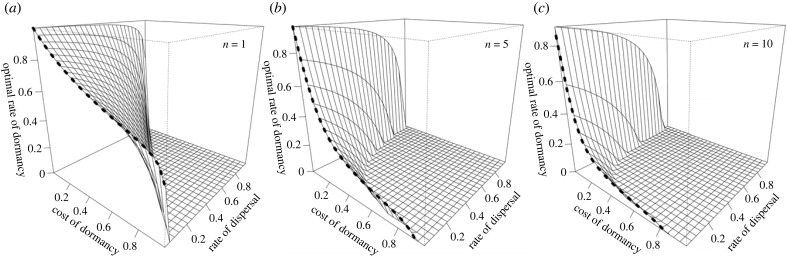
Reduced dispersal and a lower mortality cost of dormancy promote the evolution of density-independent dormancy. The optimal rate of dormancy (δ*) increases as both the mortality cost of dormancy (*c*) and the rate of dispersal (*d*) decrease, as revealed by expression (2.5) of the main text. For the purpose of illustration we assume an infinite duration of dormancy and patch size *n* = 1 (panel *a*), *n* = 5 (panel *b*) and *n* = 10 (panel *c*). Dashed lines correspond to the special case of a vanishingly low level of dispersal (*d* → 0), as given by expression (2.6) of the main text.

In this infinite-duration special case, the optimal rate of dormancy is a monotonically decreasing function of the cost of dormancy (*c*), patch size (*n*), and dispersal rate (*d*) (figure 1). The analogy between dormancy and dispersal is more closely realised in the limit of zero dispersal, as this exactly recovers the standard infinite island model of the evolution of dispersal, but for dormancy. That is, instead of individuals being able to undergo costly dispersal to a random patch infinitely distant in space, they are able to undergo costly dormancy to a random future generation infinitely distant in time. As *d* → 0, expression (2.5) becomes

2.6 $$\delta^{*}\to \frac{r-c}{r-c^{2}}=\frac{2}{1+2cn+\sqrt{1+4c^{2}(n-1)n}},$$

which represents an exact analogue of Frank's ([16], equation (2.8)) and Taylor's ([22], equation (2.8)) results for dispersal in space, but given here for dispersal in time (figure 1, dashed lines). Finally, narrowing attention to the scenario in which there is only one parent in each patch (i.e. *n* = 1) yields an exact analogue of Hamilton & May's [5] key result that the optimal probability of dispersal is 1/(1 + *c*), but for dispersal in time rather than space, such that at least half of all individuals are expected to enter dormancy even in the limit of lethal dormancy (figure 1*a*, dashed line).

### (b) The constant non-dormant principle

Crespi & Taylor [6] investigated how kin selection shapes the evolution of dispersal when patches vary in density (i.e. when different patches contain different numbers of individuals immediately prior to the dispersal phase) and individuals are able to adjust their probability of dispersal according to that density. They found that because kin competition is greater in high-density neighbourhoods, the individuals in these neighbourhoods are favoured to exhibit a higher probability of dispersal. Additionally, they found that this probability of not dispersing is inversely proportional to the density of the individual's patch, such that the absolute number of non-dispersing individuals is expected to be the same in every neighbourhood and completely independent of local density. Crespi & Taylor considered dispersal in space, and so, in order to see if the same logic holds for dispersal in time, we expand on the model of the previous section to allow for variable patch density and individuals who are able to condition their dormancy behaviour on the density of their patch.

We assume that there is vanishingly small variation in density between patches and that individuals may adjust their probability of dormancy according to the number of individuals on their patch immediately prior to the dormancy phase. Defining the relative density *P* of the individual's patch as this number divided by the average of this number across all the patches in the population, we find that if the convergence stable probability of entering dormancy takes an intermediate value, this is given by

2.7 $$\;\delta_{P}^{*}=1-\frac{1}{P}(1-\;\delta^{*}),$$

where δ* is the convergence stable probability of dormancy from the density-independent model, given by expression (2.2), and here corresponds to the probability of dormancy that is favoured for individuals in patches of average density (i.e. *p* = 1; see electronic supplementary material for details).

Thus, we find that the convergence stable probability of dormancy is an increasing function of the density of the individual's patch and, in particular, the associated probability of not going dormant is inversely proportional to the relative density of the patch (i.e. 1 − δ_P_* ∝ 1/*P*).

Rearranging expression (2.7), and making the substitution of expression (2.2), yields an expression for the absolute number of individuals who do not go dormant within a given patch, given by

**Figure 2.**
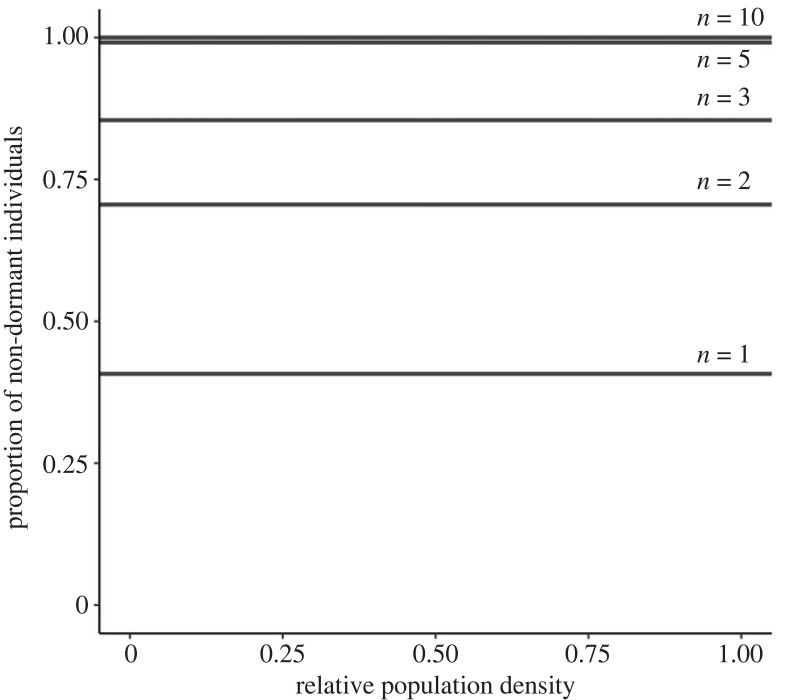
The constant non-dormant principle. When individuals are able to adjust their dormancy behaviour according to local density, they are favoured to do so, with the optimal probability of not entering dormancy being inversely proportional to patch density (i.e. 1 − δ_P_* = *k*/*P*), such that the absolute number of non-dormant individuals is completely independent of local density (i.e. *P*(1 − δ_P_*) = *k*)—as revealed by expression (2.9) of the main text. For the purpose of illustration we assume an infinite duration of dormancy, a dispersal rate of *d* = 0.1, a mortality cost of dormancy of *c* = 0.5 and a range of patch sizes *n*.

2.8 $$P(1-\;\delta^{*})=1-\delta^{*}=1-\frac{(1-d)\;r-c}{\left(1-d)(r-c^{2}\right)},$$

and which is completely independent of patch density, *P* (figure 2). This yields a direct analogue of Crespi & Taylor's ‘constant non-disperser principle’ [6,8], but for dispersal in time rather than in space. This may be termed the ‘constant non-dormant principle’.

Substituting expression (2.5) into expression (2.7) obtains the convergence stable probability of dormancy in the limit of infinitely long dormancy and no possibility of encountering genetic relatives as

2.9 $$\begin{array}{rl} & \;\delta_{P}^{*}=\\ & 1-\frac{1}{P}\left(1+\frac{2cn((1-d)-c)+(1-c(1-d))\left(1-\sqrt{1+4c^{2}(n-1)n}\right)}{2cn(1-c^{2})(1-d)}\right). \end{array}$$

Moreover, in the limit of zero dispersal (*d* → 0), equation (2.9) reduces to

2.10 $$\;\delta_{P}^{*}=1-\frac{1}{P}\left(1-\frac{2}{\;1+2cn+\sqrt{1+4c^{2}n(n-1)})}\right),$$

which represents an exact analogue of Kanwal & Gardner's [8] expression (2.7), but for density-dependent dispersal in time rather than density-dependent dispersal in space.

### (c) An invariance relationship between dormancy and altruism

Taylor [7] found that the evolutionarily favoured level of altruism in a viscous population is completely independent of the rate of dispersal, owing to the exact cancellation of the two opposing effects of dispersal in reducing relatedness and relaxing kin competition. Taylor's result concerns the dispersal of individuals in space; here, we investigate whether an analogous result is obtained for dispersal of individuals in time.

We continue to make use of the density-independent dormancy model described in section 2a, but consider that the population is monomorphic with respect to the dormancy strategy, such that all individuals undergo dormancy with probability *δ*. Additionally, we consider that there is social interaction between the *n* breeders in each patch that modulates the survival of their offspring (this is mathematically equivalent to Taylor's assumption that social interaction modulates a breeder's fecundity). Specifically, we assume that the probability of offspring survival is given by *s*(*α,β*), where *α* is the parent's level of altruism and *β* is the average level of altruism among the *n* breeders of that patch, where an offspring's survival is a decreasing function of its parent's altruism (i.e. ∂*s*(*α,β*)/∂*β* = −*C* < 0) and an increasing function of the overall altruism within the patch (i.e. ∂ *s*(*α,β*)/∂*β* = *B* > 0).

We find that the condition for natural selection to favour an increase in altruism is

2.11 $$\begin{array}{rl} & -C+Br-(B-C){\left(\frac{\left(1-d)(1-c\;\delta \right)}{1-c\;(1-d)\;\delta }\right)}^{2}\\ & \left(\;{\left(\frac{1-\delta }{1-c\;\delta }\right)}^{2}r+2\left(\frac{1-\delta }{1-c\;\delta }\right)\left(\frac{(1-c)\;\delta \;}{1-c\;\delta }\right)R+{\left(\frac{(1-c)\;\delta }{1-c\;\delta }\right)}^{2}\rho \right)>0 \end{array}$$

(see electronic supplementary material for details). This condition is again a form of Hamilton's rule and admits an inclusive fitness interpretation. An increase in a breeder's level of altruism: (i) incurs a survival cost *C* for the individual's own offspring; (ii) provides a survival benefit *B* to random offspring born in the patch, who are related to the focal individual by *r*; and (iii) leads to a net increase in *B* − *C* surviving offspring who competitively exclude their patchmates with probability ((1 − *d*)(1 − *cδ*)/(1 − *c*(1 − *d*)*δ*))^2^ and these patchmates are related to the focal individual in the usual way (see explanation of equation (2.3)).

Rearranging expression (2.11) into the form *C*/*B* < *A* yields the ‘potential for altruism’ [23],

2.12 $$\begin{array}{rl} & r\;{\left(1-c\;(1-d)\;\delta \right)}^{2}-\;{\left(1-d\right)}^{2}\\ & A=\frac{\left(r\;{\left(1-\delta \right)}^{2}+\delta \;(1-c)\;(2\;(1-\delta )\;R+\delta \;(1-c)\rho )\right)}{{\left(1-c\;(1-d)\;\delta \right)}^{2}-\;{\left(1-d\right)}^{2}}\\ & r\;{\left(1-\delta \right)}^{2}+\delta \;(1-c)\;(2\;(1-\delta )\;R+\delta \;(1-c)\rho , \end{array}$$

representing the threshold cost-to-benefit ratio below which natural selection favours an increase in altruism and below which natural selection favours a decrease in altruism. Here, the potential for altruism is expressed in terms of relatedness coefficients that are not model parameters, but rather emerge as consequences of the model's more basic demographic parameters. As in sections 2a,b, it is not possible to provide general expressions for these relatedness coefficients solely in terms of the demographic parameters. However, without any loss of generality, expression (2.3) may be substituted into expression (2.12) to obtain

2.13 $$A=\;\frac{1}{n}.$$

This reveals that Taylor's [7] cancellation result holds not only for dispersal of individuals in space but also more generally for dispersal of individuals through both space and time [7]. That is, owing to an exact cancellation of the effects of relatedness and kin competition, the potential for altruism is completely independent of both the rate of spatial dispersal and the rate of dormancy and this holds for all distributions of dormancy duration.

### (d) Density-dependent dormancy alleviates kin competition and promotes altruism

Kanwal & Gardner [8] showed that when individuals are able to adjust their probability of dispersal according to the density of their natal patch, the concomitant alleviation of kin competition promotes the evolution of altruism such that a greater degree of population viscosity is associated with a greater potential for altruism. Kanwal & Gardner's result concerns the dispersal of individuals in space, but they speculated that dormancy may act analogously to alleviate the kin-competition consequences of altruism. Here, we investigate whether an analogous result does in fact obtain for dispersal of individuals in time.

We combine the density-dependent dormancy model described in section 2b and the altruism model described in section 2c by considering a population in which social interaction between breeders modulates the survival of their offspring and individuals are able to condition their probability of dormancy on their relative patch density. Upon the assumption of vanishingly low variation in altruism, and thus vanishingly low variation in patch density, we recover the ‘constant non-dormant principle’ of section 2b and obtain the following condition for natural selection to favour an increase in altruism:

2.14 $$\begin{array}{rl} & -C\;+B\;r-(B-C)\;{\left(\frac{1-d}{1-c(1-d)\delta }\right)}^{2}(1-c)(R\;(1-\delta )\\ & \;+\rho \;\delta (1-c))-(B-C)\;(1-\delta )\frac{c(1-d)}{1-c\;(1-d)\;\delta }r>0 \end{array}$$

(see electronic supplementary material for details). Expression (2.14) is in the form of Hamilton's rule and, again, admits an inclusive fitness interpretation. An increase in a breeder's level of altruism: (i) incurs a survival cost *C* for the individual's own offspring; (ii) provides a survival benefit *B* to random offspring born in the patch, who are related to the focal individual by *r*; (iii) leads to a net increase in *B* − *C* surviving offspring who with probability ((1 − *d*)/(1 − *c*(1 − *d*)*δ*))^2^ do not disperse, and—because of the ‘constant non-dormant principle’—will all undergo dormancy, so with probability 1 − *c* they will survive dormancy and competitively exclude a future patchmate who either did not undergo dormancy with probability 1 − *δ* and hence is related to the focal individual by *R* or else did undergo—and survived—dormancy with probability *δ*(1 − *c*) and hence is related to the focal individual by *ρ*; and (iv) leads to *B* − *C* extra surviving individuals valued by *r*, a portion of which do not disperse, given by probability (1 − *d*)/(1 − *c*(1 − *d*)*δ*), and will thus undergo dormancy, and with probability *c*, these individuals will suffer the mortality cost of dormancy.

We can rearrange expression (2.14) into the form *C / B* < *A*, again giving the ‘potential for altruism’ [23],

2.15 $$A=\frac{\begin{array}{rl} & r\;{\left(1-c\;(1-d)\;\delta \right)}^{2}-c\;(1-d)\;r\;(1-\delta )\;(1-c(1-d)\delta )\\ & -{\left(1-d\right)}^{2}\;(\;(1-c)(1-\delta )\;R+\delta \;{\left(1-c\right)}^{2}\;\rho ) \end{array}}{\begin{array}{rl} & {\left(1-c\;(1-d)\;\delta \right)}^{2}-c\;(1-d)\;r\;(1-\delta )\;(1-c(1-d)\delta )\\ & -{\left(1-d\right)}^{2}\;(\;(1-c)(1-\delta )\;R+\delta \;{\left(1-c\right)}^{2}\;\rho ) \end{array}}.$$

Here, the potential for altruism is again expressed in terms of relatedness coefficients that are not model parameters, but rather emerge as consequences of the model's more basic demographic parameters. Yet, unlike the potential for altruism obtained under density-independent dormancy in section 2c, these relatedness coefficients do not vanish upon making the substitution of expression (2.3). That is, when individuals are able to condition their dormancy behaviour on relative patch density, there is no longer an exact cancellation of the relatedness and kin competition effects of dormancy, such that the overall rate of dormancy may now have some influence over the potential for altruism. No general solution obtains for the potential for altruism, so as a special case, we again consider the limit of infinitely long dormancy (i.e. such that *R* = *ρ* = 0), which yields

2.16 $$A=\;\frac{(1-(1-d)c)\;r}{1-c(1-d)(r(1-\delta )+\delta )},$$

and, upon making the substitution of expression (2.4), this becomes

2.17 $$A=\;\frac{\left(1-c\;(1-d)\;)(1-c\;(1-d)\;\delta \right)}{\begin{array}{rl} & 1+2d(n-1){\left(1-\delta \right)}^{2}-d^{2}(n-1){\left(1-\delta \right)}^{2}\\ & +(n-1)(2-\delta )\delta +c^{2}{\left(1-d\right)}^{2}\delta (1+(n-1)\delta )\;\\ & -c(1-d)(1+(2n-1)\delta ) \end{array}}.$$

**Figure 3.**
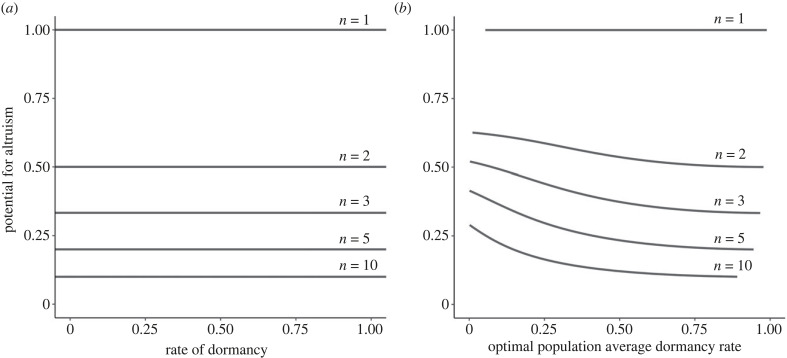
Density-dependent dormancy promotes the evolution of altruism. (*a*) The potential for altruism (*a*) is entirely independent of the rate of density-independent dormancy (*δ*), as revealed by equation (2.13) of the main text. (*b*) The potential for altruism (*a*) is a monotonically decreasing function of the overall rate of density-dependent dormancy (*δ*) when individuals employ the ‘constant non-dormant’ principle, as revealed by expression (2.17) of the main text. Both panels assume an infinite duration of dormancy, a dispersal rate of *d* = 0.1, and a range of patch sizes *n*.

In this infinitely long and density-dependent dormancy scenario, the potential for altruism is a monotonically decreasing function of the overall rate of dormancy, *δ* (figure 3*b*). This is because a reduction in the overall rate of dormancy leads to higher relatedness among patchmates, which promotes altruism, and the opposing pressure of kin competition, which inhibits altruism, is weakened owing to the competition-alleviating effect of density-dependent dormancy. This result qualitatively mirrors that of Kanwal & Gardner [8], but for density-dependent dispersal in time rather than density-dependent dispersal in space. Indeed, in the limit of zero dispersal (*d* → 0) expression (2.17) reduces to

2.18 $$A=\;\frac{1-c\;\delta }{1+\;((n-1)(2-(1+c)\;\delta )-c)\delta },$$

which is exactly equivalent to the potential for altruism reported in Kanwal & Gardner's [8] equation (2.10), but for density-dependent dispersal in time rather than density-dependent dispersal in space.

## 3. Discussion

There has been considerable research focus on the social evolutionary causes and consequences of dispersal of individuals in space. Here, we have shown that directly analogous results may be obtained for dispersal of individuals in time—that is, dormancy. First, we have demonstrated that kin selection drives the evolution of dormancy as a means to reduce competition between relatives for reproductive resources. Second, we have shown that when individuals are able to condition their probability of dormancy on local density, they are favoured to do so, such that a greater proportion of individuals are favoured to undergo dormancy in higher-density neighbourhoods and, indeed, the absolute number of non-dormant individuals is predicted to be invariant with respect to a neighbourhood's density—the ‘constant non-dormant principle’. Third, we have demonstrated that, owing to an exact cancellation of the relatedness and kin-competition consequences of dormancy, the rate of density-independent dormancy has no effect on the evolution of altruistic behaviour. Fourth, we have shown that this cancellation fails in the context of density-dependent dormancy, with the constant non-dormant principle yielding an alleviation of kin competition that leads to an overall promotion of altruism in populations exhibiting lower rates of dormancy.

We have shown that kin competition may be a powerful driver of the evolution of dormancy. In the special case of a single asexual breeder per patch, infinitely long durations of dormancy, and the absence of dispersal in space, more than half of individuals are favoured to undergo dormancy even as the associated mortality approaches certainty. This produces a direct analogue Hamilton & May's [5] classic result, but for dispersal of individuals in time rather than in space. Our analysis also reinforces previous theoretical studies that have suggested that delayed germination of plant seeds can be driven by competition among siblings in environmentally homogeneous environments [11,12,14,15]. In contrast with these previous theoretical treatments of seed dormancy, which have given analytical results only for dormancy that lasts a single generation, our analysis has yielded results for dormancy of an arbitrary length and has given particular prominence to dormancy of infinite duration, which better facilitates the comparison with dispersal in space in the classic infinite island model.

Although serving to alleviate kin competition within an individual's natal generation, dormancy may also lead to an intensification of kin competition in subsequent generations, with the inclusive-fitness consequences depending on the probability distribution of dormancy duration and the rate of dissipation of genetic relatedness over time—somewhat analogous to scenarios in which the consequences of social behaviours extend posthumously over multiple generations [24]. Indeed, if dormancy is sufficiently common and there is a substantial likelihood that two individuals entering dormancy in the same generation will also exit dormancy in the same future generation, then dormancy may act to exacerbate rather than alleviate kin competition. This explains why the convergence stable level of dormancy cannot exceed one-half when dormancy lasts only a single generation [11,12].

There is some empirical support for kin competition favouring the evolution of dormancy in the germination patterns of plant seeds. Zammit & Zedler [25] report a negative correlation between seed family size and germination fraction in the semi-aquatic *Pogogyne abramsii*. The same relationship is also found in desert annuals [26], cleistogamous annual grasses [27] and short-lived perennial desert species [28], though the latter has only marginal support. A more recent experiment on three common dryland winter annuals [29], however, suggests no significant relationship between maternal fecundity and germination fraction. There does not appear to have been any direct empirical investigation of the relationship between kin competition and diapause—a key process in the life history of insects that involves a dormant state whereby development is dramatically slowed or completely shut down [30]—but this may provide a useful future avenue for testing theory.

Insofar as individuals are able to modulate their dormancy in a density-dependent manner, our analysis suggests that a fixed absolute number of non-dormant individuals is expected in all patches, irrespective of between-patch variations in density. This constant non-dormant result mirrors Crespi & Taylor's [6] ‘constant non-disperser principle’, but applies to dispersal of individuals in time rather than in space. There is some empirical evidence that high-density environments favour the evolution of dormancy in both plant seed and diapause systems. Seed germination rates have been found to be strongly density-dependent in both annual and perennial plant species, with higher local seedling densities leading to more individuals delaying their germination [31–33]. Similarly, evidence suggests that some insects perform diapause in a density-dependent manner, especially in prolonged diapause that lasts more than a year [34]. Crowding induced diapause is well-documented in stored-product insects [35,36] and a positive relationship between rate of diapause and local larval density has also been found in treehole mosquitos [37].

We have also shown that, in the simple scenario of density-independent dormancy, the effects of increased relatedness and increased kin competition arising from a reduction in the rate of dormancy exactly cancel, such that there is no overall relationship between the rate of dormancy and the evolutionary potential for altruistic behaviour. We have obtained this result for all possible distributions of the duration of dormancy (i.e. it holds irrespective of whether dormancy lasts a single generation or a very large number of generations). This invariance result is a direct analogue of Taylor's [7] prediction that the evolutionarily favoured level of altruism is independent of the rate of dispersal of individuals in space; here, we obtain the same cancellation when considering dispersal in both space and time. There does not appear to have been any empirical investigation of the impact of dormancy rate on the evolution of altruism and this may provide another useful avenue for future tests of theory. In particular, there is growing interest in the altruistic behaviour of plants [38,39], a domain wherein dormancy is relatively common, and this suggests possibilities for comparative and experimental tests of theoretical findings. For example, stand performance—which has been used as a proxy for altruistic behaviour [38]—is an experimentally amenable as well as a socioeconomically important trait.

Taylor's [7] altruism invariance result has stimulated a great deal of theoretical and empirical interest in the role for dispersal of individuals in space to modulate social evolution, with a particular focus on identifying mechanisms that decouple the relatedness and kin-competition consequences of dispersal so that altruism may flourish in viscous populations [23,40–47]. Recently, Kanwal & Gardner [8] have shown that when individuals are able to adjust their dispersal according to local density, the resulting ‘constant non-disperser principle’ leads to a complete elimination of the kin-competition effect and an accompanying promotion of altruism in viscous populations. Here, we have found that when individuals are able to adjust their dormancy according to local density, the resulting constant non-dormant principle also leads to a complete elimination of the kin-competition effect and a concomitant promotion of altruism in viscous populations. In the special case of dormancy of infinitely long duration and vanishingly low rates of dispersal, we have obtained an exact analogue of Kanwal & Gardner's [8] key result, but for dispersal in time. The aforementioned experimental investigation of stand performance imposed in a controlled setting whereby the investigator enforces different regimes of density-dependent seed dormancy may provide one opportunity for empirical testing of this theoretical prediction.

In order to facilitate synthesis with classic results concerning the evolution of dispersal [5–8], we have assumed an infinite island model such that dispersal of individuals in space results in their travelling to effectively infinitely distant patches where no relatives are encountered. It would be useful to extend the present analysis of the social evolutionary causes and consequences of dormancy to a spatially explicit setting [47–50]. In addition, we have assumed that the cost of dormancy is entirely independent of its duration, analogous to how models of the evolution of dispersal typically assume a fixed mortality cost independent of the distance travelled, but more realistically, individuals will be less likely to survive dormancy the longer its duration. This would serve to reduce the average duration of successful dormancy, and thereby exacerbate kin competition, which is likely to make a quantitative impact—though perhaps not a qualitative impact—on the results reported here. Moreover, we have assumed that the duration of dormancy is described by a fixed probability distribution and have not considered how selection might shape this distribution, including in relation to not only expected duration but also the evolution of short-term versus long-term dormancy strategies—analogous to the distinction between short-range versus long-range dispersal [51].

Furthermore, we have followed Hamilton & May [5] in assuming a stable habitat where all patches are fully populated in every generation, such that an individual's dispersal in space or time does not alleviate competition for herself, but only for natal patchmates she leaves behind. Incorporating spatial and temporal heterogeneity in the number of breeders would be expected to yield an additional direct fitness benefit for dispersal and dormancy as a means of transporting the individual to less-competitive conditions [5]. Finally, we have assumed an asexual mode of reproduction, but it would be useful to extend the present analysis to incorporate sexual reproduction in the variety of modes exhibited by taxa that employ dormancy. Our assumption of clonal reproduction ensures that the evolutionary interests of parents and offspring are perfectly aligned, so that our results apply irrespective of whether an individual's dormancy phenotype is controlled by their own genotype or that of their parent. Yet, as with dispersal [52–57], sexual reproduction may create opportunities for parent-offspring and intragenomic conflicts in relation to dormancy [58]. Intragenomic conflicts may be especially relevant in the context of plant seeds, with imprinted genes in triploid endosperm having been shown to regulate seed dormancy [59]. Integrating these complexities into our model represents an interesting avenue for future exploration.

## Data Availability

No data were used in this paper. Supplementary material is available online [60].
